# Thyroid Function and Growth Regulation under Normal and Abnormal Conditions

**DOI:** 10.4061/2011/805036

**Published:** 2012-04-12

**Authors:** Guillermo Juvenal, Daniel Christophe, Pierre Roger, Mario Pisarev

**Affiliations:** ^1^División Bioquímica Nuclear, Comisión Nacional de Energía Atómica, Avenue del Libertador 8250, 1429 Buenos Aires, Argentina; ^2^Institut de Recherche Interdisciplinaire en Biologie Humaine et Moléculaire (IRIBHM), Université Libre de Bruxelles, IBMM, Biopark Charleroi Brussels South, 6041 Gosselies, Belgium; ^3^Institut de Recherche Interdisciplinaire, Université Libre de Bruxelles, Campus Erasme, 1070 Brussels, Belgium

The thyroid gland exerts a major control on the physiology of the whole body, and it raises fundamental questions about molecular physiological processes. Moreover, an increased proliferation of thyroid cells is associated with several pathologies, and several mechanisms may be involved. Thyroid disorders are very common, affecting millions of people. These include hypothyroidism, hyperthyroidism, thyroid nodules, thyroid cancer, and so forth, but they are also associated with other nonthyroid disorders. This special issue is devoted to illustrate the particular richness of current investigations in the field of thyroid function and growth regulation under normal and abnormal conditions.

Although thyroid gland function is mainly under the control of pituitary TSH in normal conditions, other factors may also play a role in this process. Thyroid disease is more common in women than in men. Tania Weber Furlanetto and Ana Paula Santin reviewed the direct effects of estrogens on thyroid function and growth regulation in the paper titled “*Role of estrogen in thyroid function and growth regulation.*” 

Thyroid cancer is the most common endocrine malignancy, and its incidence has significantly risen in the last decades in the world. The knowledge how thyroid cancer develops is expanding rapidly. The sequential acquisition of mutations which arise as a consequence of damage to the genome is required in order to transform a normal cell into a malignant one. The understanding of the process of thyroid carcinogenesis at the molecular level will improve not only the diagnostic but also the treatment of this pathology.

Ioannis Legakis and Konstantinos Syrigos describe the molecular events associated with the progression and dedifferentiation of thyroid carcinoma in the paper titled “*Recent advances in molecular diagnosis of thyroid cancer.*” Thyroid-specific transcription factors regulate thyroid-specific gene expression and organogenesis. Their possible role in thyroid cancer as well as in the maintenance and/or activity of stem cells is discussed by Shioko Kimura in the paper titled “*Thyroid-specific transcription factors and their roles in thyroid cancer.*” MicroRNAs (miRNAs) are short ribonucleic acid molecules (around 22 nucleotides) found in eukaryotic cells. miRNAs are posttranscriptional regulators that bind to complementary sequences on messenger RNA transcripts inducing the translational repression or messenger RNA degradation. Several miRNAs have been found to have links with some types of cancer. Francesca Marini, Ettore Luzi, and Maria Luisa Brandi reviewed the role of miRNAs in thyroid cancer development in the paper titled “*MicroRNA role in thyroid cancer development.*” Growth factors play a role in thyroid proliferation and function, while EGF acts as a mitogen for thyroid cells inhibiting also thyroid differentiation. TGF-*β* is a potent inhibitor of thyroid cell growth. However, in some transformed thyroid cells this inhibition is lost. The role of TGF-*β* and EGF on thyroid carcinogenesis and the crosstalk between these growth factors are discussed by Gabriella Mincione Maria Carmela Di Marcantonio, Chiara Tarantelli, Sonia D'Inzeo, Arianna Nicolussi, Francesco Nardi, Caterina Francesca Donini, and Anna Coppa in the paper titled “*EGF and TGF-*β*1 effects on thyroid function.*”

Grave's disease and Hashimoto's thyroiditis are the two main types of autoimmune thyroid disease. Occasionally they are also associated with other autoimmune diseases. Emina Kasumagic-Halilovic, Asja Prohic, Begler Begovic, and Nermina Ovcina-Kurtovic bring additional support to the existence of a significant association between vitiligo and thyroid autoimmunity in the paper titled “*Association between vitiligo and thyroid autoimmunity.*” 

Although some authors have found an association between an abnormal thyroid condition and bipolar disorder, little is known about the implication of the hypothalamo-pituitary-thyroid in neuropsychological deficits. Subho Chakrabarti review the last findings on this topic including genetic and neuroimaging investigations (*Thyroid Functions and Bipolar Affective Disorder*).

*Guillermo Juvenal* *Guillermo Juvenal* *Daniel Christophe* *Daniel Christophe* *Pierre Roger* *Pierre Roger* *Mario Pisarev* *Mario Pisarev*

